# Polyphenolic Compounds Extracted and Purified from *Buddleja Globosa* Hope (Buddlejaceae) Leaves Using Natural Deep Eutectic Solvents and Centrifugal Partition Chromatography

**DOI:** 10.3390/molecules26082192

**Published:** 2021-04-10

**Authors:** Jeniffer Torres-Vega, Sergio Gómez-Alonso, José Pérez-Navarro, Julio Alarcón-Enos, Edgar Pastene-Navarrete

**Affiliations:** 1 Laboratorio de Farmacognosia, Departamento de Farmacia, Facultad de Farmacia, Universidad de Concepción, Concepción PC4030000, Chile; jeniffertorres@udec.cl; 2 Regional Institute for Applied Scientific Research, Faculty of Chemical Sciences, University of Castilla-La Mancha, PC13071 Castilla-La Mancha, Spain; sergio.gomez@uclm.es (S.G.-A.); Jose.PNavarro@uclm.es (J.P.-N.); 3 Laboratorio de Síntesis y Biotransformación de Productos Naturales, Universidad del Bío-Bío, Chillán PC3800708, Chile; jualarcon@ubiobio.cl

**Keywords:** centrifugal partition chromatography, natural deep eutectic solvents, *Buddleja globosa*, Matico, phenylpropanoids, verbascoside

## Abstract

Chemical profiling of *Buddleja globosa* was performed by high-performance liquid chromatography coupled to electrospray ionization (HPLC-DAD-ESI-IT/MS) and quadrupole time-of-flight high-resolution mass spectrometry (HPLC-ESI-QTOF/MS). The identification of 17 main phenolic compounds in *B. globosa* leaf extracts was achieved. Along with caffeoyl glucoside isomers, caffeoylshikimic acid and several verbascoside derivatives (β-hydroxyverbascoside and β-hydroxyisoverbascoside) were identified. Among flavonoid compounds, the presence of 6-hydroxyluteolin-7-*O*-glucoside, quercetin-3-*O*-glucoside, luteolin 7-*O*-glucoside, apigenin 7-*O*-glucoside was confirmed. Campneoside I, forsythoside B, lipedoside A and forsythoside A were identified along with verbascoside, isoverbascoside, eukovoside and martynoside. The isolation of two bioactive phenolic compounds verbascoside and forsythoside B from *Buddleja globosa* (Buddlejaceae) was successfully achieved by centrifugal partition chromatography (CPC). Both compounds were obtained in one-step using optimized CPC methodology with the two-phase solvent system comprising ethyl acetate-n-butanol-ethanol-water (0.25:0.75:0.1:1, *v/v*). Additionally, eight Natural Deep Eutectic Solvents (NADESs) were tested for the extraction of polyphenols and compared with 80% methanol. The contents of verbascoside and luteolin 7-*O*-glucoside after extraction with 80% methanol were 26.165 and 3.206 mg/g, respectively. Among the NADESs tested in this study, proline- citric acid (1:1) and choline chloride-1, 2- propanediol (1:2) were the most promising solvents. With these NADES, extraction yields for verbascoside and luteolin 7-*O*-glucoside were 51.045 and 4.387 mg/g, respectively. Taken together, the results of this study confirm that CPC enabled the fast isolation of bioactive polyphenols from *B. globosa*. NADESs displayed higher extraction efficiency of phenolic and therefore could be used as an ecofriendly alternative to classic organic solvents.

## 1. Introduction

*Buddleja globosa* Hope (Buddlejaceae) is a native species cultivated in Chile, Peru and Argentina. In Chile *B. globosa* grows from Santiago to Patagonia [1]. It is known as ‘matico’, ‘palguin’ and ‘pañil’ and is widely used as a medicinal plant [2]. Its large, perennial leaves contain phenylpropanoids, iridoids, terpenes and flavonoids [3,4,5]. Matico is a plant often used in Mapuche culture and is applied for the treatment of different wounds (internal and external), as well as intestinal and liver problems [6,7,8]. In the previous work of Backhouse et al. [9] matico leaves were extracted with solvents of increasing polarity to obtain different fractions. These fractions were used in conjunction with a methanolic crude extract to evaluate the in vivo antinociceptive effect. In addition, verbascoside, luteolin 7-*O*-glucoside and apigenin 7-*O*-glucoside were isolated and identified. Importantly, all extracts showed an analgesic concentration-dependent effect. Verbascoside was more active than ibuprofen in the writhing test after oral administration, while luteolin 7-*O*-glucoside was more active in the tail-flick test when was used topically. On the other hand, the in vitro antioxidant effect of matico on DPPH assay and rat liver microsomes has been investigated, finding a direct relationship between antioxidant activity and the content of polyphenols determined by Folin-Ciocalteau method [10]. Since oxidative stress is a hallmark of inflammatory processes, the anti-inflammatory activity displayed by matico extracts may be explained, at least in part, in terms of their antioxidant activity. For instance, in other study of Backhouse et al. [11], a *B. globosa* extract shown scavenging properties against DPPH and superoxide anion radical. These properties were in line with the analgesic and anti-inflammatory activities linked with the inhibition of xanthine oxidase. In the same work, authors isolate beta-sitosterol, stigmasterol, stigmastenol, stigmastanol, campesterol and beta-sitosterol-glycoside, compounds that shown anti-inflammatory properties in the 12-*O*-tetradecanoylphorbol-13-acetate-induced ear edema assay. Furthermore, there have been reported that these extracts promote in vitro fibroblast proliferation, which together with the antioxidant activity may explain the wound healing properties claimed for this plant [6]. Verbascoside also known as acteoside has been pointed out as the compound responsible for most of its bioactive properties. Despite of these bioactivities are attributed to verbascoside, there are a paucity of chemical studies regarding the identification of other compounds present in this plant. Furthermore, in-depth phytochemical reports using high-end technologies are scarce for this plant. Phenolic compounds identification in plant matrix can be a complex task because there is a wide variety of structures. Besides, many polyphenol standards are not commercially available. The separation techniques that have been used to determine phenolic compounds in *Buddleja globosa* were namely, gas chromatography (GC) and high-performance liquid chromatography (HPLC), all coupled to different detection systems [11,12]. However, there is not studies considering liquid chromatography coupled to mass spectrometry. So, due to its inherent characteristics of accurate mass measurements and multiple stages analysis, the integrated strategy of liquid chromatography (LC) coupled with time-of-flight mass spectrometry (TOF-MS) and ion trap mass spectrometry (IT-MS) is well-suited to be performed as qualitative analysis tool in this field [13]. Solid support-based chromatography are the most used to purify phenolic compounds, however, irreversible adsorption and stationary phase limitations decreases the efficiency of the isolation process [14]. Centrifugal partition chromatography (CPC) is a support-free technology that has been successfully used to isolate phenylethanoid glycosides compounds [15]. Due to these advantages CPC could be used to isolate large amount of bioactive compounds to perform pharmacological assays. Until now, the isolation of bioactive compounds from *B. globosa* has been performed by conventional extraction techniques [9,16] using organics solvents such as alcohols, chloroform and ethyl acetate. However, some of these organic solvents are often toxic, flammable, explosive, and poorly biodegraded. In the last decade, several eco-friendly alternatives to the use of organic solvents have appeared. Among them, new types of solvents known as Deep Eutectic Solvents (DES) have been developed. DES are a mixture between a halide salt or other hydrogen bond acceptor (HBA) and a hydrogen bond donor (HBD) [17]. Dai and coworkers [18] reported the preparation of several DES of natural origin termed Deep Natural Eutectic Solvents (NADES). NADES solvents are obtained exclusively from natural components (e.g., sugars, organic acids, amino acids) which are commonly present in the cells of living organisms, unlike ordinary DES [19,20]. The components of NADES are characterized by the presence of several functional groups such as hydroxyls, carboxyl, or amino groups. Those groups can form intermolecular hydrogen bond, leading to highly structured viscous liquids, which accounts for their specific physical properties and different solubilizing behavior compared to conventional solvents. Those liquids can also form hydrogen bonds with solutes, thereby greatly increasing the solubility of compounds in NADES, e.g., phenolic compounds. They also show very good physicochemical properties: liquid state below 0 °C, adjustable viscosity, a broad range of polarities, and ability to dissolve a wide range of compounds [21,22]. This high solubilization strength have been reported for rutin, in some cases, as much as 12,000 times higher than water [23]. All these properties indicate their great potential as green extraction solvents for natural products [21]. In the present work, for the first time we explore eight selected NADES as extraction solvents to obtain bioactive components of *B. globosa*. Considering the above mentioned in the text, the aims of the present work were to carry out a qualitative and quantitative characterization of phenolic compounds contained in matico leaves using HPLC-DAD-IT/MS and HPLC-DAD-Q-TOF/MS, to develop a one-step separation of main *B. globosa* bioactive compounds namely, verbascoside and forsythoside B using centrifugal CPC partition chromatography (CPC) and to evaluate the feasibility of eight different NADESs for the extraction of bioactive compounds from *B. globosa* compared with 80% methanol.

## 2. Results and Discussion

### 2.1. HPLC Fingerprint Profile of Buddleja Globosa Methanol Extract

**Figure 1:**
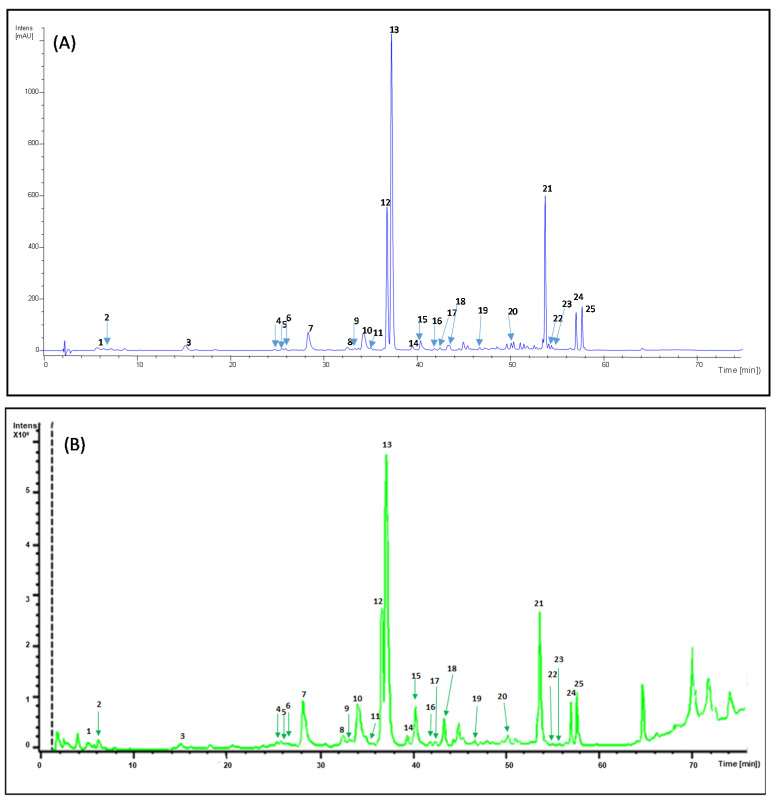
(**A**) The HPLC-UV chromatogram of *Buddleja globosa* obtained at 320 nm. (**B**) Total ion chromatogram (TIC) 100–1000 *m/z* of the 80% methanol extract of *Buddleja globosa*. The peaks numbers are the same of Table 1.

Figure 1A,B illustrate the HPLC-DAD-MS chromatograms of phenolic compounds of a *B. globosa* extract obtained with 80% methanol (soxhlet procedure). Peaks were identified with numbers (1–24) according to the elution order.

As summarized in Table 1 and Table 2, chromatographic analysis enabled the identification of 17 compounds, while 8 remains unknown. Assignments were based on UV-visible bands and MS data, including experimental and calculated *m/z* for provided assumed formulas, errors and the main fragments obtained by MS-MS and identified compounds for each peak. All the compounds were identified by their QTOF-MS and the MS-MS spectra acquired with the IT-MS and data provided by the literature.

**Figure 2:**
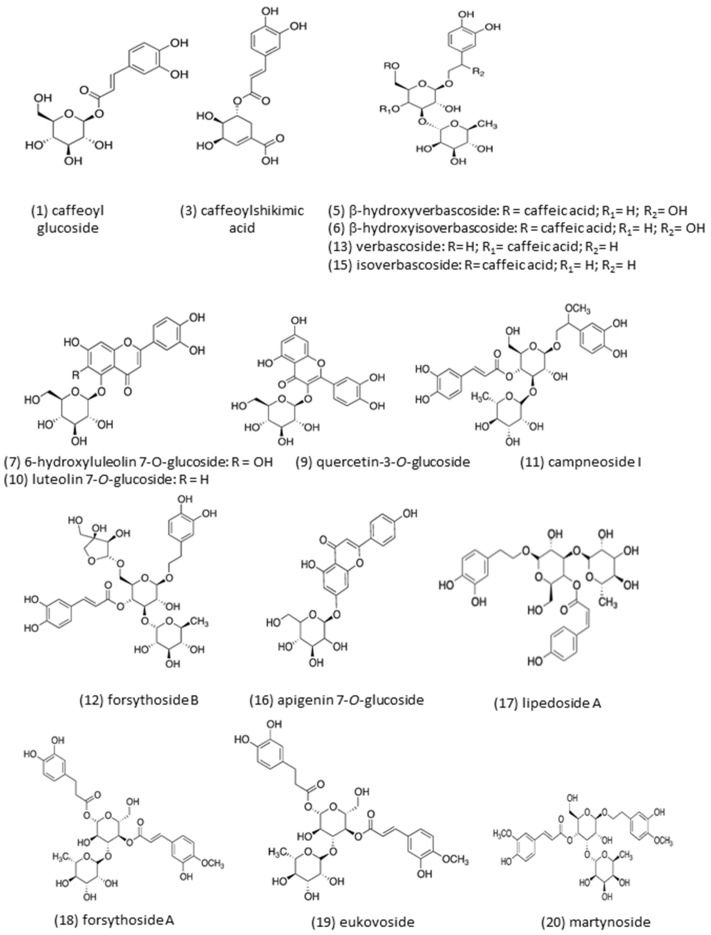
Chemical structures of the identified compounds in *Buddleja globosa.*

**Figure 3:**
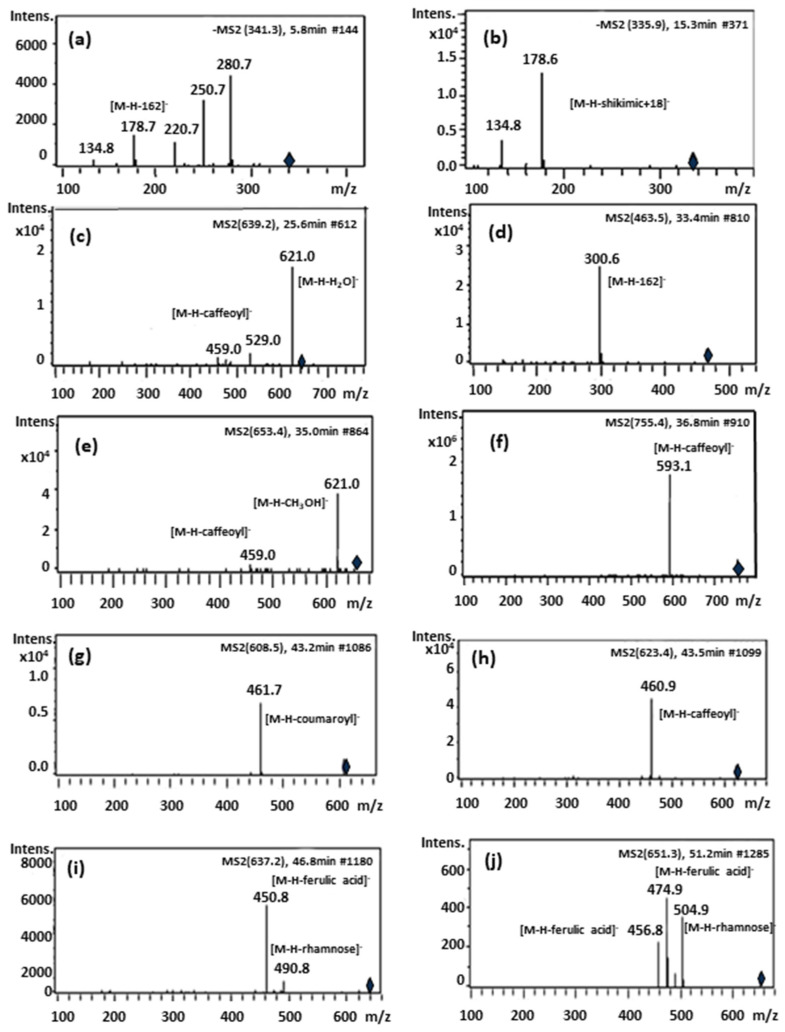
Most representative MS-MS spectra of *Buddleja globosa* compounds no reported previously. The spectra correspond to: (**a**) caffeoylglucoside, (**b**) caffeoylshikimic acid, (**c**) β-hydroxy-verbascoside or β-hydroxy-isoverbascoside, (**d**) quercetin-3-*O*-glucoside, (**e**) campneoside I, (**f**) forsythoside B, (**g**) lipedoside A, (**h**) forsythoside A, (**i**) eukovoside and (**j**) martynoside.

Figure 2 shows the chemical structures of the identified compounds in *B globosa*. Overall, caffeoyl derivatives, phenylpropanoid glycosides and flavonoids were the most dominant compounds present in *B. globosa*. Compound 1 (Rt = 5.61 min) and compound 2 (Rt = 7.19 min) showed similar molecular ions [M-H]^−^ at *m/z* 341.3 and 341.0, respectively. The MS-MS spectrum of both compounds yielded fragment ions at *m/z* 281, 251, 221, 179 and 135 (Figure 3a). The fragment ion at *m/z* 179 [M-H-162]^−^ correspond to caffeic acid which suffer a neutral loss of glucose moiety (162 Da). For compounds 1 and 2 in negative ionization mode, the molecular formulas of ions at *m/z* 342.09569 and 342.09544 were predicted as C_15_H_18_O_9_.This data are consistent with the identity assignation previously reported [24], where these compounds were tentatively identified as isomers of caffeoyl glucoside. To the best of our knowledge this is the first report of these compounds in *B. globosa*. Compound 3 (Rt = 15.14 min) was identified as caffeoylshikimic acid (C_16_H_16_O_8_, *m/z* 336.2129). This compound has characteristic UV max at 296 and 326 nm and give rise to a molecular ion [M-H]^−^ at *m/z* 335.6 with ion fragments MS-MS at *m/z* 178.6 and 134.8 (Figure 3b). The fragment ion at *m/z* 178.6 [M-H − C_7_H_10_O_5_ + 18] suggest the loss of dehydrated shikimic acid, while fragment ion at *m/z* 134.8 indicate an additional loss of -CO_2_ (44 Da). However, esterification site (for 5-, 4- or 3- derivatives) in this compound could not be determined in the present study [25]. This compound is reported in *B. globosa* leaves for the first time herein compounds 5 (Rt = 25.49 min) and 6 (Rt = 25.89 min) produced molecular ions [M-H]^−^ at *m/z* 639. 2 and ion fragments at *m/z* 621, 529 and 459. Fragment at *m/z* 621 [M-H_2_O]^−^ in MS-MS experiment belong to the loss of water (H_2_O), fragment ion at *m/z* 529 correspond to the loss of catechol unit and fragment ion at *m/z* 459 corresponded to the loss of the caffeic acid moiety (Figure 3c). For compounds 5 and 6 in negative ionization mode, the molecular formulas of ions at *m/z* 640.20136 and 640.20113 were predicted as C_29_H_36_O_16_. These compounds were tentatively identified by comparison of its MS-MS fragmentation pattern to literature data [26], being coherent with β-hydroxyverbascoside diastereoisomers. It is important to mention that both compounds have not been reported in previous studies regarding *B. globosa*.

Compounds 7 (Rt = 28.32 min) and 9 (Rt = 33.30 min), showed identical molecular ions at *m/z* 463. MS-MS from precursor ion *m/z* 463 yield an abundant ion fragment at *m/z* 301 in MS–MS [M–H–162], suggesting the loss of a hexose unit. For compound 7 in negative ionization mode, the molecular formula of ions at *m/z* 464.09707 was predicted as C_21_H_20_O_12_. Retention time and UV spectra indicates that compound 7 is 6-hydroxyluteolin-7-*O*-glucoside, a compound reported previously by Hougthon and Mensah [27]. For compound 9 in negative ionization mode, the molecular formula of ions at *m/z* 464.09569 was predicted as C_21_H_20_O_12_. The UV spectrum, retention time and literature data [28], obtained for compound 9, tentatively suggest that this flavonoid could be quercetin-3-*O*-glucoside (isoquercitrin).

Compound 10 (Rt = 34.26 min) produce a molecular ion [M-H]^−^ at *m/z* 447.2 yielding in MS-MS experiment an ion fragment [M–H–162] at *m/z* 284.6 suggesting a neutral loss of glucose. This compound was identified as luteolin 7-*O*-glucoside by comparison of their retention time, UV and MS-MS spectra with those of reference standard. For compound 10 in negative ionization mode, the molecular formula of ion at *m/z* 448.10213 was predicted as C_21_H_20_O_11_. This flavonoid was also been reported in *B. globosa* leaves [9,16,26].

Compound 11 (Rt = 35.02 min), produce a molecular ion [M-H]^-^ at *m/z* 653 yielding in MS-MS experiment an ion fragment at *m/z* 621 [M-H-methoxy]^-^ and other at *m/z* 459 [M-H-methoxy- caffeoyl]^-^ corresponding to the loss of the caffeoyl moiety from the main ion fragment at *m/z* 621 [26]. In negative ionization mode, the molecular formula of ions at *m/z* 654.21747 was predicted as C_30_H_38_O_16_. This compound was tentatively identified as Campneoside I, which is reported for the first time in *B. globosa*.

Compound 12 (Rt = 36.76 min) exhibited a pseudo-molecular ion [M–H]^−^ at *m/z* 755, showed an intense signal from ion fragment at *m/z* 593 due to a loss of a caffeoyl moiety (Figure 3f). In negative ionization mode the molecular formula of ions at *m/z* 756.2506 was predicted as C_34_H_44_O_19_. This compound was identified as forsythoside B according with literature data [29,30].

Compound 13 (Rt = 37.23 min) and Compound 15 (Rt = 40.37 min) exhibited a pseudo-molecular ions [M–H]^−^ at *m/z* 623. The ion fragments at *m/z* 461 [M−H–caffeoyl]^−^ corresponded to the loss of the caffeoyl moiety. In negative ionization mode the molecular formulas of ions at *m/z* 624.20799 and 624.20749 were predicted as C_29_H_36_O_15_. Based on comparison of their retention times, UV spectra and MS-MS fragmentation pattern compounds 13 and 15 were identified as verbascoside and its structural isomer, isoverbascoside. Verbascoside represent the dominant peak present in the extract of *B. globosa* already reported [9,16].

Compound 16 (Rt = 40.61 min) exhibited a pseudo-molecular ions [M–H]^−^ at *m/z* 431.2. The ion fragments at *m/z* 268.7 [M−H–162]^−^ corresponded to the neutral loss of glucose. Based on its MS-MS fragmentation pattern, compound 16 was identified as apigenin 7-*O*-glucoside. In negative ionization mode the molecular formula of ions at *m/z* 432.10609 was predicted as C_21_H_20_O_10_. This assignation was confirmed by comparison of its retention time, UV and MS-MS spectra with those of reference standard. This compound was previously reported by Backhouse and coworkers [11]

Compound 17 (Rt = 41.85 min) shown a pseudomolecular ion at *m/z* 607.5 [M-H]^−^ and ion fragment in MS-MS experiment at *m/z* 460.9 [M-H-coumaroyl] corresponding to a loss of a coumaroyl (−146 Da) moiety. In negative ionization mode the molecular formula of ions at *m/z* 608.17317 was predicted as C_28_H_32_O_15_.This compound was tentatively identified as lipedoside A [31]. To the best of our knowledge this is the first report of this compound in *B. globosa*.

Compound 18 (Rt = 43.45 min) exhibited a pseudo-molecular ions [M–H]^−^ at *m/z* 623.4. The ion fragments at *m/z* 460.9 [M−H–caffeoyl]^−^ corresponded to the loss of the caffeoyl moiety. According to this MS-MS profile, compounds 18 is another verbascoside isomer (Table 1 and Table 2). In negative ionization mode the molecular formula of ions at *m/z* 624.20729 was predicted as C_29_H_36_O_15_. This compound was identified tentatively as forsythoside A since showed UV bands and MS-MS profile in line with data provided in literature [25,26,32].

Compound 19 (Rt = 46.70 min) exhibit a pseudo-molecular ions [M–H]^−^ at *m/z* 637.3. This molecular ion show a deprotonated molecular ion 14 Da higher than verbascoside, corresponding to the presence of a methyl group in the molecule. In MS-MS experiment, an ion fragment due to loss of rhamnose moiety [M-H-rhamnose] is observed at *m/z* 491. Another ion fragment at *m/z* 461 is due to loss of feruloyl group [M-H-ferulic acid]^−^. In negative ionization mode the molecular formula of ions at *m/z* 638.22184 was predicted as C_30_H_38_O_15_. Therefore, compound 19 was tentatively identified as the verbascoside derivative eukovoside [13,26,33].

Compound 20 (Rt = 51.04 min) exhibit a pseudo-molecular ion at *m/z* 651. The ion fragments at *m/z* 505 [M-H-rhamnosyl]^−^ represent the neutral loss of rhamnose, while ion fragments at *m/z* 475 and 457 represented the neutral loss of feruloyl unit and water, respectively. In negative ionization mode the molecular formula of ions at *m/z* 652.23711 was predicted as C_31_H_40_O_15_. These data suggest that compound 20 is martynoside [26]. This is the first report for this compound in *B. globosa*.

Compound 21 (Rt = 53.73 min) exhibit a pseudo-molecular ion at *m/z* 763 and MS-MS fragments at *m/z* 667.8 and 548.6 corresponding to simultaneous loss of 96 and 120 Da due to partial cross-ring cleavage observed in C-glycosides. Additionally, the ion fragment at *m/z* 488.9 is originated by the loss of 60 Da is observed in the cross-ring cleavage of C-pentosides [34,35]. The loss of 187 Da that generate an ion fragment at *m/z* 301.7 could not be rationalized.

### 2.2. Isolation of Main Polyphenols from B. globosa by Centrifugal Partition Chromatography:

The nine two-phase solvent systems listed in Table 3 were selected from literature and tested with the aim to separate the major target compounds in *B. globose* extract, namely forsythoside B and verbascoside (compounds 12 and 13) [15,32,36,37,38,39]. The most suitable KD values to perform the CPC isolation process were obtained with the solvent systems based on ethyl acetate-n-butanol-ethanol-water. Calculated KD’s values for compounds 12 (forsythoside B) and 13 (verbascoside) with the solvent system 1 were 0.51 and 3.02, respectively.

**Figure 4:**
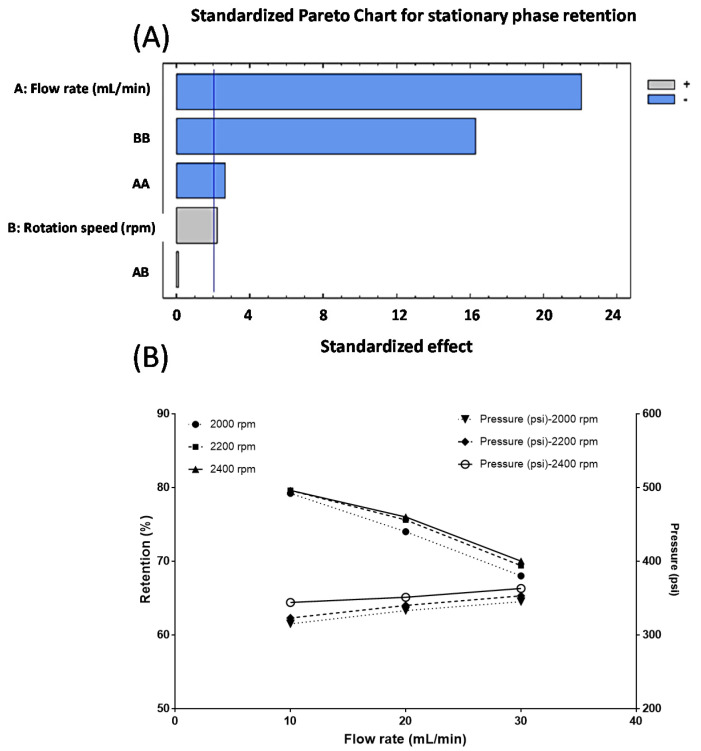
(**A**) Pareto chart showing the standardized effect of independent variables on the stationary phase retention response. (**B**) Effect of flow rate and rotation speed on stationary phase retention and backpressure.

**Figure 5:**
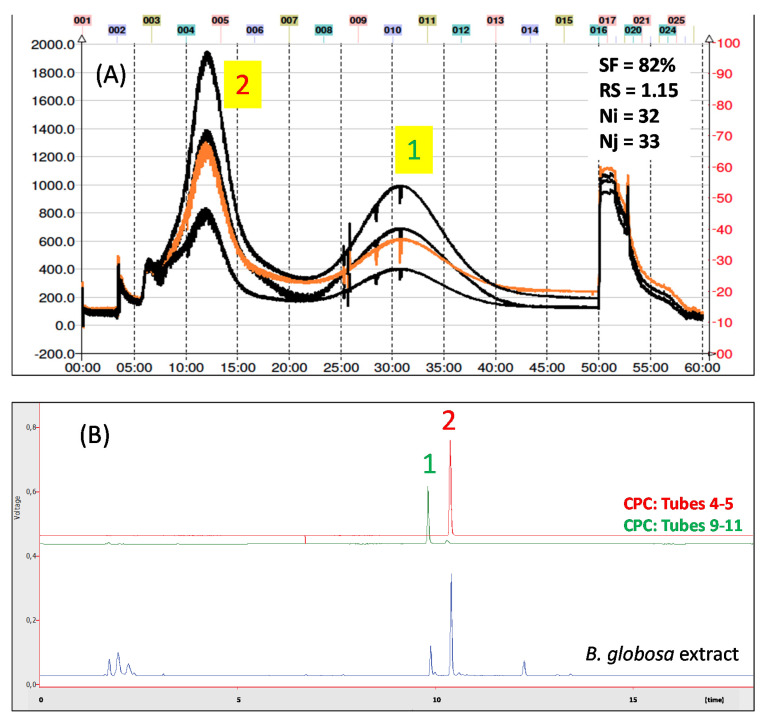
(**A**) CPC trace of *Buddeja globosa* extract. Inset: experimental stationary retention (SF); resolution (RS), and theoretical plates (N i,j) for target compounds 1 and 2 after pulse injection under optimal separation conditions. (**B**) HPLC of forsythoside B (1, green trace) and verbascoside (2, red trace) isolated from *Buddleja globosa* by CPC in ascending mode.

According with these results, in ascending mode compound 13 will elute first, whereas compound 12 should elute at higher retention time. In order to optimize the CPC separation process we investigate the effect of rotation speed and flow rate upon the retention of stationary phase inside CPC rotor. So, the Pareto diagram (Figure 4A) shows the influence of each of the variables studied (flow rate and rotation speed) on the percentage of stationary phase retention (response). Pareto ranking analysis showed that the most significant variables that negatively influence the stationary phase retention response were the flow rate and the quadratic factor of rotation speed. Accordingly, from this diagram it can conclude that the most important variable that significantly affects (*p* < 0.05) stationary phase retention is the flow rate. This parameter affect negatively, since greater flow rate values results in poor stationary phase retention. Figure 4B illustrate the variation in the stationary phase retention and backpressure responses as function of: flow rate and rotation speed. Therefore, from this diagram it can conclude that the most important variable that significantly affects (*p* < 0.05) stationary phase retention is the flow rate. This parameter affect negatively, since greater flow rate values results in poor stationary phase retention. As shown in Figure 4B, the variation in the stationary phase retention and backpressure responses are functions of: flow rate and rotation speed. In this graph the term flow rate is linear, therefore the optimum conditions for this variable is located in some point between the range 5–10 mL/min and 2000–2400 rpm for the rotation speed. Hence, we select 7.5 mL/min and 2200 rpm as work conditions since the retention of stationary phase was 82%. Under these conditions, pressure remained in 320–360 psi and the time elapsed to obtain the target compounds did not exceed 60 min. As shown in Figure 5A, two well-defined peaks are observed at 12 and 32 min, respectively. The number of theoretical plates (N) for both compounds was similar, considering that the separation was carried out in the CPC extractor model, in which normally lower N values are obtained compared to the results observed in CCC or CPC equipment. The latter device has twin-cells of smaller size and in greater number (e.g., SCPC-250) than the SCPC-B Bio-Extractor used in the present work [40]. However, the resolution (RS) between both compounds was 1.15, which is considered sufficient to obtain a preparative separation and subsequent scaling. The KD values calculated under the optimized conditions for compounds 1 and 2 were 1.15 and 0.32, respectively. These values did not matched those obtained in the initial studies with the shake flask method used to select the solvent system from Table 3. The difference observed for such values could be due to the type of method used. In the shake flask procedure, an amount of 5 mg is dissolved in 3 mL of the biphasic system, while in the determination of KD by CPC, the pulse injection of 1 mL implies that the sample volume is only <1% of the total volume of CPC rotor. Therefore, the injection volume does not influence the main parameters that govern the separation by CPC. The partition coefficient is not constant since it depends on the temperature and the pressure and above all on the concentration in a two-phase system. In our conditions, the volume of the stationary phase at equilibrium (SR) is 82%. That means that 173 mL correspond to stationary phase and 38 mL to mobile phase. Therefore, both compounds are highly diluted after a pulse injection, which allows us to state that the calculated KD´s are closer to the real values [41,42]. After several CPC runs, the HPLC analysis of the core cuts from the center of the peaks allow to confirm the presence of verbascoside and forsythoside B in high purity (Figure 5B).

### 2.3. Comparison of the Extractability of Phenolic Compounds with NADES.

Table 4 shows the list of eight NADES solvents used in this study. Comparing with traditional organic solvents, the main drawback of NADES is their high viscosity, which causes slow mass transfer and results in a decrease in their extraction ability. In order to solve this problem, it was necessary adding water (20%) to improve polyphenol diffusion [43]. In a recent study of our group, the incorporation of 20% water improved the extractability of phenolic compounds and alkaloids from *Peumus boldus* using different NADES [44]. However, the percentage of water should not exceed certain limits since it could alter the structure of the supramolecular network of NADES and reduce the extraction of more lipophilic compounds [45,46]. Also, other study showed that increasing NADESs temperature leads to a decrease in their viscosity [47]. For such reason, in the present work a temperature of 60° C was selected. Another way to increase mass transfer and to speed up the diffusion rate of compounds in NADES is to apply external forces such as stirring. Stirring is the simplest way to speed up the diffusion rate of the compounds in the liquid.

**Figure 6:**
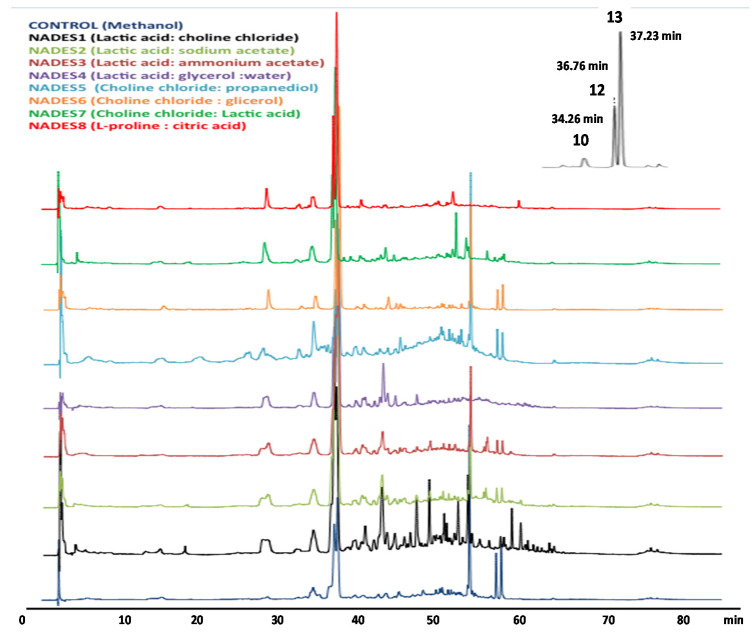
HPLC-UV chromatogram (320 nm) of phenolic compounds from *Buddleja globosa* leaves extracted with different NADES solvents. Peaks 10 (Rt = 34.26 min), 12 (Rt = 36.76 min), and 13 (Rt = 37.23 min) correspond to luteolin 7-*O*-glucoside, forsythoside B and verbascoside, respectively.

**Figure 7:**
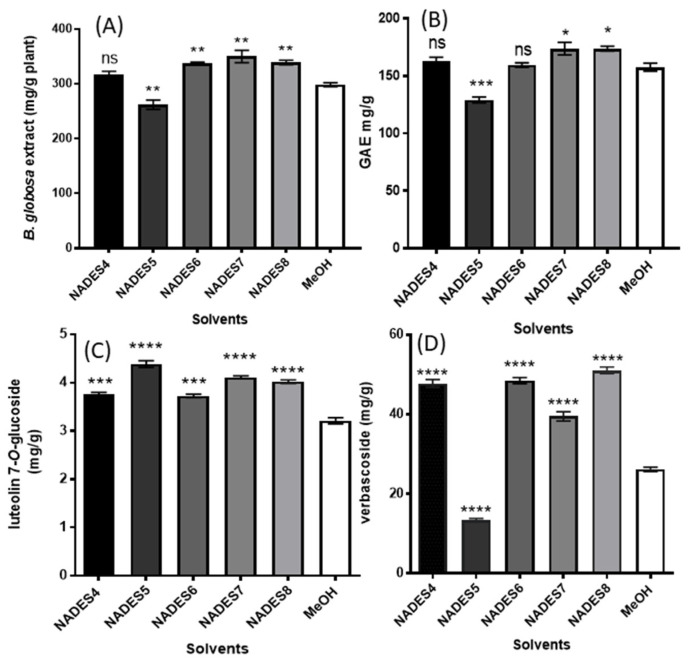
Effect of different NADESs on the extraction yields of *B. globosa* (**A**); Total Polyphenols (**B**); 7-*O*-glucoside (**C**), and verbascoside (**D**) from *Buddleja globosa* leaves. Data correspond to the means ± SD (n = 3). There is a significant difference when * *p* < 0.05; **p < 0.01; *** *p* < 0.001 or **** *p* < 0.0001 versus 80% methanol. Abreviation: ns: not significant.

To compare the extraction efficiencies of the NADESs with those obtained from traditional organic solvents, extractions with a mixture of MeOH: water (80:20, *v/v*) were performed in parallel under the same extraction conditions and analyzed by HPLC-DAD-ESI-IT-MS (Figure 6). After extraction, the extraction yields (mg/g of dried plant) was determined after drying the extractable polyphenols and the total phenolic content (TP) was performed using the Folin-Ciocalteau reagent (Figure 7A,B). Polyphenol extraction and extract yields show a similar trend as seen in Figure 7. However, NADES4 and 6 did not show significant differences in terms of their efficiency to extract polyphenols compared to 80% methanol. Only NADES5 had a significantly lower performance than 80% methanol, while NADES7 and 8 were the best in this determination. However, quantitative analysis of verbascoside and luteolin 7-*O*-glucoside only was possible for NADESs 4, 5, 6, 7 and 8 as presented in Figure 7C,D, whereas in NADES 1, 2 and 3 this analysis was not possible due to the poor separation of forsythoside B and verbascoside. The result obtained for luteolin 7-*O*-glucoside (Figure 7C) using NADESs 4, 6, 7, and 8 shown that a significantly highest amount of such compound can be extracted in comparison with 80% MeOH. With this organic solvent, 3.206 mg/g of luteolin 7-*O*-glucoside was determined, whereas choline chloride-1, 2 propanediol (NADES5), increase the extraction yield up to 36.8% (4387 mg/g of luteolin 7-*O*-glucoside). This result agreed with those reported by García and coworkers [48], who found that 1,2-propanediol-based NADESs are excellent extraction solvents for flavonoids. These authors state that such NADES are more efficient for the extraction of polyphenols from olive oil due to their lower viscosity. Meng and colleagues [49], reported that choline chloride and 1,2-propanediol at 1:4 molar ratio allows an improved extraction of quercetin, naringenin, kaempferol and isorhamnetin from Pollen Typhae. On the other hand, the results obtained for verbascoside (Figure 7D) with NADESs 4, 6, 7 and 8 shown that a significantly highest amount of this polyphenol was extracted in comparison with 80% MeOH (26.165 mg/g) used as control organic solvent. Importantly, NADES based on proline and citric acid (NADES8) enable a two-fold increase of extracted compounds (51.045 mg/g), while no significant differences (*p* < 0.0001) were found regarding to lactic acid/glycerol/water (NADES4) and choline chloride/glycerol (NADES6), obtaining 47.771 and 48.474 mg/g, respectively. This latter result suggest that NADES4 could be also used as alternative. Ivanović et al. [50], reported a yield of 14.23 mg/g of verbascoside in *Lippia citriodora* using choline/lactic acid NADES. These results agree with those published in previous studies with organic acid-based NADESs, which confirm that this class of solvent can effectively facilitate the extraction of phenolic bioactive compounds [51]. Our results are consistent with these findings and explain the higher efficiency of NADES 4, 6 and 7. NADES5 showed a similar efficiency with the other NADES with respect to the group of flavonoids (represented by luteolin 7-*O*-glucoside). Nevertheless, despite its low viscosity, NADES5 had a very poor efficiency on verbascoside extraction. Furthermore, in previous works [52] the reported viscosity of this NADES was 31.6 cp, which is even lower than that of NADES6 prepared with glycerol (47 cp). This latter finding suggests that viscosity is not the only physicochemical property that define the extraction efficiency of a NADES. One of the most important factors for efficient extraction is related to the molecular structure of NADES, since its polarity must be as close as possible to that of the target molecule. It is possible that the polarity of NADES5 is different from that of verbascoside but very close to that of luteolin 7-O-glucoside. However, according to the previously published data, the NADES5 and 6 should have similar polarity (~56 kcal/mol), determined as molar transition energy (ENR) using the solvatochromism of dye Nile red [53,54]. Hence, the extraction of verbascoside should be similar for both NADES. Another possible explanation for this difference relates to the ratio between donor and acceptor (HBA/HBD) NADES components. If the ratios between donor and acceptor in NADES 5, 6 and 7 are observed in Table 4, NADES5 is the one that has the lowest proportion of choline chloride, and thus provide a lesser amount of chloride ions. This fact not only affects the viscosity of NADES but also its basicity, since if there is less choline, there will be less interaction with the weak acidic hydroxyl groups of the verbascoside. In addition to the abovementioned, NADES6 has glycerol with three –OH groups, while NADES5 has 1,2 propanediol with two –OH, having lower capacity to form hydrogen bonds [49].

## 3. Materials and Methods

### 3.1. Plant Material

Leaves of *B. globosa* were collected in Chiguayante, Bío-Bío Region, Chile in April, 2017 and their voucher specimens, (CONC N° 187540) were identified by Dr. Roberto Rodriguez, and deposited in the Herbarium of the Botany Department, University of Concepción, Chile. Firstly, leaves were air-dried at room temperature in the dark for 14 days, and then ground to a fine powder using a domestic blender (Bosch MMB 112R, Stuttgart, Germany). This material was used for all further procedures. For LC-MS analyses, extraction of phenolic compounds was carried out using a soxhlet system. In brief, 25 g of the pulverized material was weighed, and 100% methanol was used as solvent. The process was carried out until the exhaustion of the plant material was produced (16 h). Then, methanol was removed under vacuum (<40 °C) to dryness. Extract was dispersed in 1 mL of 20% methanol and filtered (MFS-25, 0.22 μm TF, Whatman, Ohio, OH, USA), before being injected into the HPLC systems.

### 3.2. Chemicals and Reagents

Choline chloride, L-(+)-lactic acid, glycerol, 1, 2-propanediol were purchased from Sigma-Aldrich (Steinheim, Germany). Citric acid and L-proline were purchased from Merck (Darmstadt, Germany). Verbascoside, luteolin 7-*O*-glucoside, apigenin 7-*O*-glucoside and gallic acid commercial standards of HPLC grade (>99%) (Extrasynthese, Genay, France) were used as references for identification. Methanol, ethanol, chloroform, ethyl acetate, n-butanol, methyl tert-butyl ether and acetonitrile were of HPLC quality. Formic acid was of analytical grade (>99%).

### 3.3. Instruments and Chromatographic Conditions

#### 3.3.1. Qualitative and Quantitative HPLC-DAD-IT-MS/MS Analysis

The samples were analyzed by HPLC-DAD-ESI-MS-MS in an Agilent 1100 Series system (Agilent, Waldbronn, Germany) equipped with a DAD (G1315B) and LC/MSD Trap VL (G2445C VL) ESI-MS-MS system, and it was coupled to an Agilent Chem Station (version B.01.03) data-processing station (Favre et al. 2018). The mass spectral data were processed with the Agilent LC/MS Trap software 5.3 (version 3.3). A Zorbax Eclipse XDB-C18 Narrow-Bore column (2.1 mm × 150 mm; 3.5 μm) thermostated at 40 °C was employed and the chromatographic conditions were as follows: solvent A (water/formic acid/acetonitrile, 87:10:3, *v/v/v*), solvent B (acetonitrile/water/formic acid, 50:40:10, *v/v/v*), and solvent C (methanol/water/formic acid, 90:1.5:8.5, *v/v/v*). The flow rate was 0.190 mL min^−1^. The linear solvent gradient was as follows: zero min, 96% A and 4% B; 8 min, 96% A and 4% B; 37 min, 70% A, 17% B, and 13% C; 51 min, 30% A, 40% B, and 30% C; 56 min, 10% A, 50% B, and 40% C; 62 min, 30% B and 70% C; 68 min, 30% B and 70% C; 70 min, 100% C; 75 min 100% C and post time of 8 min 96% A and 4% de B and the injection volume was 20 μL. The mass spectrometer was run in the negative ion mode with following parameters: the capillary voltage was set at 3500 V, drying gas flow N_2_, 8 mL/min; drying temperature, 350 °C; nebulizer, 40 psi; and scan range, 100–1000 *m/z*. The results were expressed as milligrams per gram of extract (mg g^−1^ extract). The linearity of the method was assessed from the correlation coefficients (R^2^) of three set of calibration curves obtained for seven levels of verbascoside concentrations ranging from 0.5–250 mg/L (y = 152.41× − 71.644; R^2^ = 0.9998) and for luteolin 7-*O*-glucoside 0.3–22 mg/L (y = 264.73× − 51.693; R^2^ = 0.9999). Each point was injected three times. Limit of detection (LOD) were estimated at signal to noise (S/N) ratios of 3:1 and 10:1, respectively. With this procedure, LOD and LOQ values for verbascoside were 0.12 mg/L and 0.34 mg/L, respectively. For luteolin-7-*O*-glucoside, LOD and LOQ were 0.25 mg/L and 0.66 mg/L, respectively.

#### 3.3.2. Q-TOF High-Resolution Mass Spectrometry Measurements

The analytical system used consisted of a 1260 Infinity high performance liquid chromatography system coupled to a diode array detector (DAD) and a 6545 quadrupole-time of flight (Q-TOF) mass spectrometer detector (Agilent, Waldbronn, Germany). The control software was Mass Hunter Workstation (version B.06.11). The Q-TOF used a Dual Jet Stream Electrospray Ionization (Dual AJS-ESI) source operated in the negative ionization mode and the following parameters were set: capillary voltage, 3500 V; fragmentor voltage, 200 V; gas temperature, 350 °C; drying gas, 8 L/min; nebulizer, 40 psig; sheath gas temperature, 400 °C; sheath gas flow, 12 L/min; acquisition range, 100–1000 *m/z*; and CID, linear range of 30–45. Samples were analyzed after injection (10 μL) on a Zorbax Eclipse Plus C18 Rapid Resolution HD column (2.1 mm × 50 mm, 1.8 μm) protected with a 5mm guard column of the same material thermostated at 40 °C. The solvent system was water with 0.1% formic acid (solvent A) and acetonitrile-methanol (70:30, *v/v*) with 0.1% formic acid (solvent B). The elution gradient was (time, % of solvent A): 0 min, 95%; 1 min, 95%; 30 min, 65%; 35 min, 30%; 40 min, 20%; 45 min, 95%; and a post time of 8 min. Compounds were identified using the algorithm “Find by Formula” that evaluated the mass accuracy together with the isotopic relative abundance and isotopic separation.

### 3.4. Centrifugal Partition Chromatography (CPC)

#### 3.4.1. General Procedure

The centrifugal partition chromatography (CPC) device was a SCPC-250-B Bio-Extractor (SCPE) (Armen, France). Total cell volume was 250 mL. Descending and ascending modes were selected by a four-way switching valve. SCPE was connected to a SPOTPREP II system (Armen, France), equipped with an UV detector and fraction collector (32 mL tubes) and an injection valve with 10 mL sampling loop (Note: for pulse injections 1 mL loop was used). The CPC rotor was first filled with 1.5 column volumes using the lower phase at 30 mL/min and 500 rpm rotation. Afterwards, upper phase was pumped into the system in ascending mode at a flow rates from 5–40 mL/min increasing the rotation speed up to 2000–2400 rpm. Crude *B. globosa* extract (300 mg) was dissolved in 10 mL of 1:1 mixture of upper and lower phase and loaded through 10 mL sample loop. Fractions (25 mL, 26 tubes) were collected, and monitored with a scan of 200–600 nm and wavelengths 280 and 320 nm. Extrusion was performed after 50 min run time with 100% stationary phase increasing the flow rate at 30 mL for 10 min. Pareto chart was used to evaluate the effect of rotation speed and flow rate upon stationary phase retention. The final operation conditions were chosen considering the retention of stationary phase, backpressure in the CPC column and time elapsed to obtain the target compounds.

#### 3.4.2. KD, Ni, and RS Calculations

The partition coefficient (KD) was determined according to Ito and coworkers [14], with slight modifications. In brief, five mg of *B. globosa* extracts were weighed and dissolved in 3 mL of thoroughly pre-equilibrated upper organic and lower aqueous phases. In this work, nine different solvent system were selected from the list presented in Table 3. The mixture was vigorously shaken in a 10 mL conical vials. Once settled, upper and lower phases were separated and taken to dryness. The residues were reconstituted in 1 mL of mobile phase and analyzed by HPLC following the method described above, injecting 5 µL. Based on the ratio of HPLC peak area of each target compound in lower and upper phases the KD values were calculated as follows:

(1) $$\mathrm{KD}=\frac{\mathrm{HPLC}\;\mathrm{peak}\;\mathrm{area}\;\mathrm{of}\;\mathrm{target}\;\mathrm{compound}\;\mathrm{in}\;\mathrm{upper}\;\mathrm{phase}}{\mathrm{HPLC}\;\mathrm{peak}\;\mathrm{area}\;\mathrm{of}\;\mathrm{target}\;\mathrm{compound}\;\mathrm{in}\;\mathrm{lower}\;\mathrm{phase}}$$

Also, the partition coefficients of target compounds were calculated from the CPC chromatogram using pulse injection (1 mL) of *B. globosa* extract at optimal flow rate (F) according to the following Equation:

(2) $$\mathrm{KD}=\;\frac{F\;Rt,\;i-\left(1-\mathrm{SF}\right)\mathrm{VC}}{\mathrm{SF}\;\mathrm{VC}}$$

where F is the optimized flow rate used in the present study, *Rt*,*i* is the retention time of *B. globosa* target compound, SF is the stationary phase retention and VC is the column volume [40].

The number of theoretical plates (Ni) were calculated as follows [40]:

(3) $$Ni=\;{\left(\frac{Rt,i}{\sigma i}\right)}^{2}$$

where *Rt*,*i* is the retention time of the target compound *i* and *σ* is the variance of the peak.

The resolution (RS) was calculated using the following equation [40]:

(4) $$RS=2\frac{\left(Rt,j-Rt,i\right)}{wj+wi}$$

where *Rt*,*j* and *Rt*,*i* are the retention times for the second and the first target compounds, respectively. Peak widths at base line are denoted as *wj* and *wi*.

### 3.5. Preparation of NADES

The synthesis of all NADES used in the present work was based on previous research [20,51]. In brief, lactic acid, choline chloride and L-proline (hydrogen bond donor —HBD) were mixed with sodium acetate, ammonium acetate, glycerol, 1, 2-propanediol (hydrogen bond acceptors —HBA) at proper molar ratios and the mixtures were heated under stirring, until a homogeneous liquid was reached. NADES were kept in the dark inside capped flasks at ambient temperature. The codes of the NADES used in this study, along with details regarding their synthesis are shown in Table 4.

#### Extraction of Phenolic Compounds of B. globosa Leaves with Different NADES

All NADES were used as 80% (*v/v*) aqueous solutions, incorporating water 20% to reduce the viscosity. Extractions were carried out according to a previously described methodology [44,51]. Plant material (0.1 g) was placed in a 50 mL conical tube and 10 mL of NADES was added. As control, we use methanol under the same extraction conditions used for the NADES. The mixtures were shaken vigorously manually for a few seconds to form a slurry and then extracted through heating and stirring (Büchi Syncore Polyvap R24, Switzerland). Extraction conditions were: 340 rpm, 60 °C for 50 min. Then, samples were centrifuged at 8000 rpm for 10 min (Eppendorf 5804 R, USA). The supernatants were diluted four times with mobile and filtered through a 0.22 μm cellulose acetate membrane filter prior to analysis. Extraction yields, were determined after recover the sample extract from the NADES. So, the solvent was removed using solid-phase extraction (SPE) on HLB cartridges and following the method of Lui and coworkers [55] with slight modifications. In brief, HBL cartridge were equilibrated with 5 mL of methanol, followed by 5 mL of water. After loading the extract solution (2 mL), the cartridge was subsequently rinsed with 8 mL of water twice and then eluted with 8 mL of methanol in pre-weighted vials. Methanol was evaporated in heating block under nitrogen stream to obtain the extracts. Yields per gram of dry extract were calculated as follows:

(5) Y=50 x m

where *Y* is the yield and m is the mass in milligrams of dry extract.

The yield of Total Polyphenols (TP) was determinate using an adapted Folin-Ciocalteau procedure [56]. In brief, 20 μL of properly diluted samples were mixed with 780 μL of distilled water and 50 μL of Folin-Ciocalteu reagent in 1.5 mL vials. After 1 min, 150 μL of 7.5% sodium carbonate solution were added and mixed. After incubation in the dark (25 °C) for 1 h, aliquots of 200 μL were loaded in 96 well microplates. Absorbances were measured at λ 750 nm using a microplate reader (EPOC, Biotek). The analysis were performed in triplicate and normalized against negative controls (distilled water or diluted NADES) according to the Table 4 and expressed as mg of gallic acid equivalents (GAE) per gram of dried extract. The equation of gallic acid calibration curve was y = 0.089x + 0.0221 (R^2^ = 0.9981).

### 3.6. Statistical Analysis

Statistical comparison was performed using GraphPad Prism 5. (GraphPad Software, San Diego, CA, USA) Data were analyzed by a one-way analysis of variance (ANOVA) and statistical significance level was considered with **** *p* < 0.0001, *** *p* < 0.01 and * *p* < 0.05.

## 4. Conclusions

In this work, advanced analytical methods have been used to carry out a thorough characterization of a *B. globosa* extracts using on trap (IT) and time-of flight (TOF) mass analyzers. In this report, a total of 17 compounds were identified. These compounds included 13 phenylpropanoids and 4 flavonoid glycosides. To the best of our knowledge, caffeoylglucoside isomers, caffeoylshikimic acid, β-hydroxy-verbascoside, β-hydroxy-isoverbascoside, quercetin-3-*O*-glucoside, campneoside I, forsythoside B, lipedoside A, forsythoside A, eukovoside and martynoside were identified for the first time in *B. globosa* using liquid chromatography coupled with IT and TOF. Altogether, our features indicate that the strategy of coupling LC with IT-MS and TOF-MS is a powerful tool for the qualitative analysis of complex samples. Furthermore, we achieve the fast one-step purification of the two main bioactive compounds from *B. globosa* using CPC. Finally, from our results it can be concluded that NADESs are a potential green alternative to conventionally used organic solvents as extraction media for phenolic compounds. Among the NADESs tested in our study, proline— citric acid (1:1) was the most promising solvent, attaining higher extraction yields of verbascoside from *B. globosa* leaves. In the case of luteolin 7-*O*-glucoside, NADESs prepared with choline chloride-propanediol (1:2) enable greater extraction yield than samples extracted with 80% methanol. Therefore, due to its superior extraction efficiency for phenolic compounds and reduced environmental and lower economic impacts, NADESs have a great potential as green alternatives to organic solvents for the extraction of plant bioactive metabolites with medical applications.

## Figures and Tables

**Table 1:** Identification of targeted phenolic compounds from *Buddleja globosa* by LC-IT-MS-MS.

Peak	Rt (min)	[M-H]^−^ *m/z*	MS-MS Fragments	λ Max (nm)	Proposed Compound
1	5.61	341.3	280.7, 250.7, 220.7, 178.7, 134. 7	228, 294 sh, 327	Caffeoyl glucoside (isomer 1)
2	7.19	341.0	280.7, 250.7, 220.6,178.6, 134.6	296 sh, 326	Caffeoyl glucoside (isomer 2)
3	15.14	335.6	178.6, 134.8	296 sh, 326	Caffeoylshikimic acid
4	24.72	537.3	518.9, 342.2, 294.7, 234.7, 178.7	294 sh, 321	Unknown 1
5	25.49	639.2	621.0, 529.0, 459.0	296 sh, 327	β -hydroxy-verbascoside
6	25.89	639.3	621.0, 528.9, 459.1	296 sh, 329	β -hydroxy-isoverbascoside
7	28.32	463.2	300.6	255 o 258 sh, 281, 344	6-Hydroxyluteolin7-O-glucoside
8	32.49	463.4	285.3	261 sh, 281 sh, 347	Unknown 2
9	33.30	463.5	300.6	256, 260 sh, 283 sh, 351	Quercetin-3-*O*-glucoside
10	34.26	447.2	284.6	255 sh, 265, 282 sh, 346	Luteolin 7-*O*-glucoside
11	35.02	653.2	620.9, 459.0	296 sh, 332	Campneoside I
12	36.76	755.2	593.1	232, 294 sh, 331	Forsythoside B
13	37.23	623.3	460.9	296 sh, 330	Verbascoside
14	39.47	653.3	623.8, 490.8, 376.8, 308.7, 252.7	296 sh, 329	Unknown 3
15	40.37	623.2	460.8	290 sh, 327	Isoverbascoside
16	40.61	431.2	268.7	266, 333	Apigenin-7-*O*-glucoside
17	41.85	607.5	460.9	296 sh, 326	Lipedoside A
18	43.45	623.4	460.9	290 sh, 326	Forsythoside A
19	46.70	637.3	490.8, 460.8, 314.8	290 sh, 327	Eukovoside
20	51.04	651.3	504.9, 474.9, 456.8, 372.8, 329.0, 250.6,	286 sh, 329	Martynoside
21	53.73	763.4	667.8, 548.6, 488.9, 301.7	312	Unknown 4
22	54.08	785.2	738.9, 678.9, 576.8, 546.9, 462.7	296 sh, 322	Unknown 5
23	54.38	785.4	738.9, 678.9, 576.9, 547.0, 505.0, 462.9,	296 sh, 324	Unknown 6
24	57.03	755.5	709.0, 649.0, 546.9, 517.0, 433.0	298, 312	Unknown 7
25	57.67	755.4	709.0, 649.0, 546.9, 517.0, 432.9	298 sh, 314	Unknown 8

**Table 2:** Identification of targeted phenolic compounds from *Buddleja globosa* by LC-ESI-QTOF/MS-MS.

Peak	Formula	Experimental (Observed) Mass	Mass (Monoisotopic Mass) Calculated	Error ppm	[M-H]^−^ *m/z*	MS-MS Fragments	Proposed Compound
1	C_15_H_18_O_9_	342.09569	342.09508	1.78	341.08548	281.06520, 221.04528, 179.03544, 135.04563	Caffeoyl glucoside (isomer 1)
2	C_15_H_18_O_9_	342.09544	342.09508	1.06	341.08499	281.06582, 179.03449, 135.04463	Caffeoyl glucoside (isomer 2)
3	C_16_H_16_O_8_	336.2129	336. 2087	1.2	335.17819	179.03503, 135.04486	Caffeoylshikimic acid
4	C_25_H_30_O_13_	538.1693	538.16864	1.22	537.1617	537.16155, 459.14979, 399.12976, 309.06201	Unknown 1
5	C_29_H_36_O_16_	640.20136	640.20034	1.6	639.19359	621.18255, 529.15682, 459.15179, 251.05644,	β -hydroxy-verbascoside
6	C_29_H_36_O_16_	640.20113	640.20034	1.25	639.19374	621.18123, 529.15366, 459.15003, 325.09311, 251.05530	β -hydroxy-isoverbascoside
7	C_21_H_20_O_12_	464.09707	464.09548	3.43	463.08839	301.03568	6-Hydroxyluteolin 7-*O*-glucoside
8	C_21_H_20_O_12_	464.09547	464.09548	0.02	463.07781	282.06828	Unknown 2
9	C_21_H_20_O_12_	464.09569	464.09548	0.21	463.0885	300.02774	Quercetin-3-*O*-glucoside
10	C_21_H_20_O_11_	448.10213	448.10056	3.5	447.0949	285.04091	Luteolin 7-*O*-glucoside
11	C_30_H_38_O_16_	654.21747	654.21599	2.26	653.20999	621.07113, 459.12421	Campneoside I
12	C_34_H_44_O_19_	756.2506	756.24768	3.86	755.24353	593.21042	Forsythoside B
13	C_29_H_36_O_15_	624.20799	624.20542	4.12	623.20069	461.16809, 315.10869, 161.02544	Verbascoside
14	C_30_H_38_O_16_	654.21716	654.21599	1.79	653.20931	377.12629, 249.07689, 163.04006	Unknown 3
15	C_29_H_36_O_15_	624.20749	624.20542	3.31	623.20047	461.16773, 315.10891, 161.02550	Isoverbascoside
16	C_21_H_20_O_10_	432.10609	432.10565	1.03	431.09884	268.03810	Apigenin-7-*O*-glucoside
17	C_28_H_32_O_15_	608.17317	608.17412	1.57	607.16510	461.07231	Lipedoside A
18	C_29_H_36_O_15_	624.20729	624.20542	2.2	623.19644	461.16656	Forsythoside A
19	C_30_H_38_O_15_	638.22184	638.22107	1.21	637.21464	461.16669, 315.10963, 175.04031	Eukovoside
20	C_31_H_40_O_15_	652.23711	652.23672	0.59	651.23043	505.08127, 475.06172, 456.15642	Martynoside
21	C_35_H_40_O_19_	764.25426	764.25314	1.36	763.18271	668.02938, 549.15312, 489.07663, 301.81022	Unknown 4
22	C_35_H_46_O_20_	786.25933	786.25824	1.38	785.25227	623.18693, 547.17931, 463.16063, 378.91956, 291.08289, 207.06629,	Unknown 5
23	C_35_H_46_O_20_	786.25931	786.25824	1.35	785.25239	547.18298, 463.16087, 341.09857, 207.06621, 163.03947	Unknown 6
24	C_34_H_44_O_19_	756.25029	756.24768	3.46	755.2433	709.23749, 465.02019, 405.01723, 341.00981, 285.04084	Unknown 7
25	C_34_H_44_O_19_	756.25026	756.24768	3.42	755.24323	709.23553, 593.13026, 541.03515, 497.02419,	Unknown 8

**Table 3:** Partition coefficients (K_D_) of compounds 12 and 13 in two-phase solvent systems.

#	Solvent System	Ratio *v/v*	12 ^a^	13 ^a^	Ref.
1	Ethyl acetate-*n*-butanol-ethanol-water	0.25: 0.75: 0.1: 1	0.51	3.02	[32]
2	Ethyl acetate-*n*-butanol-ethanol-water	0.5: 0.5: 0.1: 1	0.19	4.96	[36]
3	Ethyl acetate-*n*-butanol-ethanol-water	4: 0.6: 0.6: 5	0.08	1.93	[36]
4	Ethyl acetate- *n*-butanol-water	10: 6: 15	1.33	0.33	[15]
5	Ethyl acetate- *n*-butanol-water	2:1:3	1.94	0.24	[37]
6	Ethyl acetate- *n*-butanol-water	13: 3: 9	0.01	0.35	[38]
7	Ethyl acetate-water	1: 1	0.00	0.02	[38]
8	Chloroform- *n*-butanol-methanol-water	3: 2: 4: 5	37.42	3.51	[39]
9	Chloroform- *n*-butanol-methanol-water	4: 3: 4: 5	41.86	3.50	[39]

^a^ KD obtained from HPLC analysis described in Section 3.4.2.

**Table 4:** Composition of the natural deep eutectic solvents (NADES) used in the present study.

Code	NADES Composition	Molar Ratio	Conditions
NADES1	Lactic acid: choline chloride	3:1	15 min; 50 °C; 700 rpm
NADES2	Lactic acid: sodium acetate	3:1	15 min; 50 °C; 700 rpm
NADES3	Lactic acid: ammonium acetate	3:1	15 min; 50 °C; 700 rpm
NADES4	Lactic acid: glycerol: water	3:1:3	40 min; 50 °C; 900 rpm
NADES5	Choline chloride: 1,2- propanediol	1:3	20 min; 60 °C; 1000 rpm
NADES6	Choline chloride: glycerol	1:2	60 min; 80 °C; 1000 rpm
NADES7	Choline chloride: lactic acid	1:1	60 min; 60 °C; 1000 rpm
NADES8	L-Proline: Citric acid	1:1	120 min; 80 °C; 1000 rpm

## Data Availability

Not applicable.

## Sample Availability

Samples of the compounds isolated in this research are available from the authors.
